# Increasing Incidence of Zygomycosis (Mucormycosis), France, 1997–2006

**DOI:** 10.3201/eid1509.090334

**Published:** 2009-09

**Authors:** Dounia Bitar, Dieter Van Cauteren, Fanny Lanternier, Eric Dannaoui, Didier Che, Francoise Dromer, Jean-Claude Desenclos, Olivier Lortholary

**Affiliations:** Institut de Veille Sanitaire, Saint Maurice, France (D. Bitar, D. Van Cauteren, D. Che, J.-C. Desenclos); Université Paris-Descartes, Paris, France (F. Lanternier, O. Lortholary); Institut Pasteur, Paris (E. Dannaoui, F. Dromer, O. Lortholary); Hôpital Georges Pompidou, Paris (E. Dannaoui).

**Keywords:** Zygomycosis, fungi, immunocompromised, mucormycosis, France, at-risk patients, research

## Abstract

We analyzed hospital records to provide a population-based estimate of zygomycosis incidence and trends over a 10-year period at a national level in France. Data showed an increasing incidence from 0.7/million in 1997 to 1.2/million in 2006 (p<0.001). We compared our data with those from the French Mycosis Study Group, a recently established voluntary network of French mycologists coordinated by the National Reference Center for Mycoses and Antifungals. We documented that incidence of zygomycosis increased, particularly in patients with hematologic malignancies or bone marrow transplants. The role of previous exposure to antifungal drugs lacking activity against zygomycetes could explain this increase but does not appear exclusive. Incidence also increased in the population of patients with diabetes mellitus. We conclude that observed trends reflect a genuine increase of zygomycosis cases in at-risk populations.

## Abstract 2

Results were derived from a population-based study using hospital discharge data.

Zygomycoses are severe angioinvasive infections caused by common filamentous fungi, the zygomycetes. These ubiquitous opportunistic fungi can cause infections with high lethality in immunocompromised or diabetic patients. Whatever the route of infection (inhalation of airborne spores, ingestion, or direct skin inoculation), the hyphae invade blood vessels, causing tissue infarction and necrosis (*1*–*4*). In healthy persons, innate immunity is sufficient to prevent infection, except in cases of massive contamination after traumatic inoculation of contaminated soil (*5*). Patients with phagocytic dysfunctions caused by neutropenia or ketoacidosis, as well as patients with high iron serum concentrations, are at high risk of developing zygomycosis (*4*). These underlying conditions can influence clinical presentation and outcome (*2*,*6*,*7*). The rhinocerebral presentation is the most frequently reported localized symptom followed by pulmonary, cutaneous, cerebral, gastrointestinal, and disseminated infections (*3*,*8*). In the rhinocerebral or pulmonary forms, patient death rates are reported to be as high as 60% because of delayed diagnosis or delayed therapeutic management (*7*–*9*). Treatment strategies are based on high doses of any lipid formulation of amphotericin B, associated with large surgical resections when possible. Most triazole agents are not effective in vivo (*10*), except for posaconazole, which shows some efficacy both experimentally and in patients as second-line therapy (*11*–*13*). The clinical contribution of new iron chelating agents remains controversial (*14*,*15*).

Some reports have suggested an increasing incidence of zygomycosis on the basis of analysis of data from monocentric studies (*7*,*10*,*14*,*16*). Several explanations have been posited: longer survival of persons with severe hematologic malignancies or solid organ transplantations; increased knowledge of the infection and thus a better diagnosis yield; and finally, prolonged use of new antifungal drugs ineffective against zygomycetes as prophylactic or empiric treatment in bone marrow transplant recipients or other immunocompromised patients (*10*,*17*–*20*). However, the incidence of infection among the general population and infection trends over time has rarely been documented at a national level. To describe the demographic characteristics of patients and to estimate the incidence and case-fatality ratio (CFR) associated with the major underlying diseases, we analyzed the electronic hospitalization and death records of patients in France in whom zygomycosis had been diagnosed.

## Material and Methods

### Data Sources

To describe cases and to estimate incidence, we extracted all hospital records related to zygomycosis from 1997 through 2006 in metropolitan France, which includes mainland France and the island of Corsica. The French hospital information system, Programme de Médicalisation du Système d’Information (PMSI), is a managerial tool based on the systematic collection of standardized administrative and medical information for any new hospital admission. An estimated 95% of all public and private hospitals use this tool, including all third-level structures, i.e., university hospitals and other reference hospitals (*21*). An anonymous extraction of the dataset with limited sociodemographic information (age, sex, and residence area) can be made available for specific epidemiologic studies. Medical information includes the main cause of admission (principal diagnosis), the related medical conditions (with >20 entries for various associated diagnoses), duration of stay, modes of admission and discharge, and major medical activities performed during the stay. Diseases are coded according to the International Classification of Diseases, 10th revision (ICD-10). This coding system was stable during the study period. Conversely, the codes for medical or surgical procedures, determined at the national level, have evolved over time, with notable changes occurring in 2003.

To estimate the overall CFR, deaths that occurred during hospitalization, including those among readmitted patients, were identified through the PMSI*.* To take into account deaths that occurred after discharge*,* we also used data from death certificates available at the institution in charge of recording and analyzing death certificates, the CepiDc. The certificate is completed at the time of death by the attending physician (at hospital or home) who records the main cause of death (i.e., the direct, immediate cause), as well as underlying diseases that may have contributed to death. The certificate is then sent to the CepiDc, where it is coded according to ICD-10.

### Case Identification

We used all PMSI records in which the ICD-10 codes B46 or B460 to B469 were identified; these corresponded to any clinical presentation of zygomycosis, or mucormycosis. To distinguish first admissions from rehospitalizations, we created a unique patient identifier by chaining the variables year of birth (derived from the patient’s age and the date of admission), gender, and residence postal code. The same identifier was used to match PMSI and CepiDc data, to identify additional deaths and to check for duplicates. However, the CepiDc identifier was based on the exact year of birth, but in the PMSI we derived the year of birth from age at admission.

### Case Definitions

We defined as unique cases those that had an identifier detected only 1 time in the PMSI during the 10-year period. Cases detected >1 time were classified as new cases at first occurrence in the database; subsequent ones were coded as readmissions. After checking for duplicates, we analyzed unique or new cases for incidence.

The zygomycosis cases were classified according to their clinical presentation, when available. When several body localizations were reported, we selected the most severe one, either in a single stay or in subsequent admissions. For instance, the association of unspecified localization (B46, B468, or B469) or gastrointestinal (B462) or cutaneous (B463) localizations, together with a pulmonary (B461), a rhinocerebral (B462), or a disseminated (B464) localization, was classified under 1 of the 3 latter categories.

We similarly analyzed the underlying diseases potentially associated with zygomycosis and focused on known risk factors. We combined the ICD-10 codes with medical procedures because some conditions, such as bone marrow transplantation, were coded as a procedure and not as a disease*.* To take into consideration patients with >1 underlying condition, we introduced a hierarchy. We first created the bone marrow transplantation (BMT) category, which included autologous and allogenic BMT, with or without graft versus host disease. To reduce underreporting bias, we looked for BMT occurrence in both incident and readmitted patients. Because of changes in the coding system regarding medical procedures, identification of autologous versus allogenic BMTs before 2003 was not possible. In a second category labeled hematologic malignancies, we included neutropenia or aplastic anemia, acute lymphoid or myeloid leukemia, lymphomas, and other disorders of the myeloid system. The third category, labeled nonhematologic immunodepression, included solid organ cancers, HIV infection, solid organ transplantations, and/or rejection of these transplantations. By convention, BMT patients (with or without graft versus host disease) who also had a neutropenia were classified under the BMT category. Finally, we analyzed diabetes types 1 or 2 patients under a separate category; for patients who had both an immunosuppressive condition and diabetes, the immunosuppressive condition was kept as the main risk factor.

### Data Analysis

Taking into account population growth, we used the 1999 national population census to estimate annual incidence rates. To enable trends comparisons, we used the same approach for the specific incidence rates in patients with underlying factors (zygomycosis patients presenting these underlying factors in the numerator and total population in the denominator). Because of heterogeneous age distributions in the population according to geographic areas, standardization of incidence rates by gender and age groups was performed in each of the 96 districts (departments) of metropolitan France. Populations in the districts ranged from 73,500 to >2.5 million inhabitants.

For CFR, we considered a possible underreporting in the PMSI combined with inaccuracies in the registration of causes of deaths in the CepiDc. To estimate the number of deaths that could have been missed by both data sources, we performed a capture–recapture analysis following the method synthesized by Gallay et al. (*22*). In a 2 × 2 table, the numbers of deaths identified in one of the respective databases (n_1,2_ or n_2,1_, in which the subscript 1 indicates identified and 2 indicates not identified) or in both databases (n_1,1_) are entered in the respective 2 × 2 table’s boxes. Under the hypothesis that both sources are independent, deaths that could have been missed by both sources (n_2,2_) can be estimated by the equation (n_2,2_ = n_1,2_ × n_2,1_ / n_1,1_). The total number of deaths is N = n_1,1_ + n_1,2_ + n_2,1_ + n_2,2_. The variance and 95% confidence intervals (CIs) are Var(N) = (n_1,1_ + n_2,1_)(n_1,1_ + n_1,2_)(n_1,2_)(n_2,1_)/(n_1,1_)^3^ and 95% CI = N ± 1.96Var(N), respectively.

The CFR estimates were impaired by the unavailability of accurate information about underlying medical conditions for deaths identified in the CepiDc*.* In addition, the approximation of the year of birth when using the PMSI dataset limited the CFR studies by age groups. We therefore restricted the analysis to the overall CFR by underlying diseases whenever possible and made no further assumptions on missing data.

### Statistical Analysis

Statistical analysis was performed by using Excel (Microsoft Corporation, Redmond,, WA, USA) and STATA-9 (StataCorp LP, College Station, TX, USA) software. The Fisher exact or χ^2^ tests were used where appropriate to compare groups. Trends were assessed by using a Poisson regression. Where appropriate, we present results with 95% CIs and p values, considering p<0.05 as significant.

## Results

### Increased Incidence of Zygomycosis in Metropolitan France

**Figure 1:**
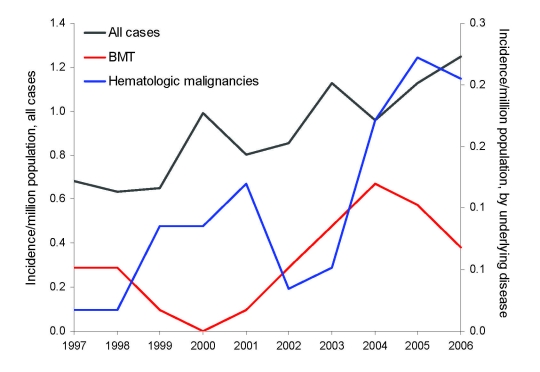
Evolution of the incidence of zygomycosis, France, 1997–2006. BMT, bone marrow transplantation.

**Figure 2:**
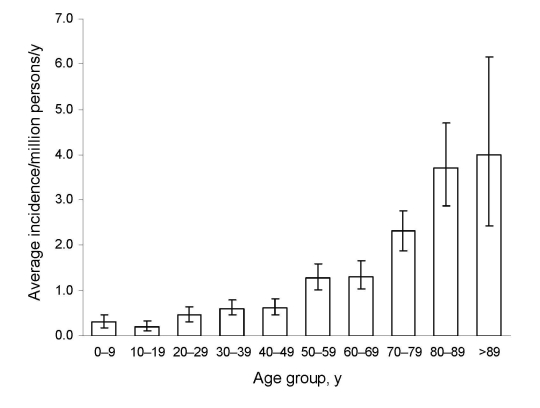
Average annual incidence rate of zygomycosis, by age group, France, 1997–2006. Error bars indicate 95% confidence interval.

Of 828 hospital stays linked to zygomycosis, 531 incident cases were identified in public and private hospitals of metropolitan France from 1997 through 2006 There were 283 males and 248 females (sex ratio 1.1); mean age was 57.1 years (median 60 years, range: <1 month–96 years). The annual incidence rate (AIR) increased from 0.7 cases/million persons in 1997 to 1.2/million persons in 2006 (Figure 1); yearly increase was +7.4% (p<0.001). Averaged over the 10 years of study, the AIR was 0.9/million persons (95% CI 0.8–1.0). This average AIR increased with age from 0.3/million in children aged 0–9 years to 3.9/million in patients >89 years of age (Figure 2).

The number of patients per district of residence varied from 0 to 36 over the 10-year period. The average standardized incidence rate varied from 0 to 3.4/million persons per district per year. The highest rates were observed in 5 rural districts hosting <500,000 inhabitants each (p<0.01): from 2.4/million (95% CI 2.3–2.5) to 3.4/million (95% CI 3.2–3.5) versus 0.9/million nationwide. In the greater Paris area hosting ≈10 million inhabitants in 8 districts, the average standardized incidence rate was 1.1/million per year (95% CI 0.9–1.2), not significantly different from the rate for the nation.

Underlying diseases among the 531 zygomycosis case-patients comprised 156 patients with immunosuppressive conditions: 33 patients with BMT, 59 patients with hematologic malignancies, and 64 patients with nonhematologic immunodepression (Table). Of the 104 patients with diabetes, 17 also experienced an underlying immunosuppressive condition and were classified as such. In zygomycosis patients with hematologic malignancies, the specific AIR increased over time from 0.02 to 0.2 cases/million persons from 1997 through 2006 (+24%/year; p<0.001) (Figure 1). It also increased in patients with BMT (+15%/year; p = 0.02) or with diabetes (+9%/year; p = 0.02). Incidence was not significantly altered over time for patients with nonhematologic immunodepression and in patients with no known risk factors (data not shown).

**Table:** Distribution of zygomycosis cases and deaths, by underlying disease, France, 1997–2006

Known risk factors	No. cases	No. deaths	Case-fatality ratio, %*
Bone marrow transplantation	33	12	36.4
Hematologic malignancies	59	21	35.6
Neutropenia/aplastic anemia	23	11	47.8
Acute lymphoid/myeloid leukemias	20	6	30.0
Other lymphoid/myeloid disorders	16	4	25.0
Nonhematologic immunodepression	64	8	12.5
Cancers of the solid organs	28	3	10.7
HIV/AIDS	26	1	3.8
Solid organ transplantations	10	4	40.0
Diabetes	86	8	9.3
No known risk factor	289	12	4.2
Total	531	61	11.5

*Calculated only for the 61 deaths reported in hospital records for which information about underlying diseases was available.

### Clinical Presentation

The reported presentations included pulmonary (18.5%), rhinocerebral (11.8%), cutaneous (9.4%), and disseminated (6.8%) localizations. The digestive tract was mentioned as a unique localization for 123 (23.2%) patients. No information was available for 30.3% of patients.

### Deaths

Sixty-one deaths were recorded in the PMSI dataset, including 7 deaths reported among readmitted patients. We identified 31 additional deaths in the CepiDc*,* resulting in a total of 92 deaths during the study period and an overall CFR of 17.3% (95% CI 9.1–31.5). Lethality evolved over time and increased from 2004 onward: average CFR was 13.7% in the 1997–2003 period versus 23.6% in the 2004–2006 period (p<0.01). Using the capture–recapture method, the overall expected number of deaths would reach 143 (95% CI 0–40) for a CFR of 26.9% (95% CI 0–100%). The specific CFR according to underlying conditions was calculated only for the 61 deaths reported in the PMSI for which information was available (Table). It was greater when patients had hematologic malignancies (47.8%) or BMT (36.4%). In patients with nonhematologic immunodepression, diabetes, or no known risk factors, the CFRs were 12.5%, 9.3%, and 4.2%, respectively.

## Discussion

### Incidence and Trends

This retrospective analysis of hospital records provides a population-based estimate of zygomycosis incidence and trends over a 10-year period at a national level. It shows an increasing incidence from 0.7/million to 1.2/million population (p<0.001). The average 0.9/million annual incidence rate in France is lower than the 1.7/million incidence reported in the population-based study by Rees et al. in California in 1992–1993 (*23*). However, our results are based on a passive routine system, whereas an active laboratory-based surveillance was implemented in the San Francisco Bay area at a time of high HIV prevalence; this system had smaller sample sizes (2.9 million in San Francisco vs. ≈60 million in France). In a survey performed in Spain in 2005, incidence was 0.43/10^6^ on a representative sample of 50 participating hospitals covering one third of the country’s population, i.e., ≈14 million inhabitants (*24*).

We compared our data with those from the French Mycosis Study Group, a recently established voluntary network of French mycologists coordinated by the National Reference Center for Mycoses and Antifungals (NRCMA). This network, which covers all university and other tertiary level hospitals in France, reported an average of 17 cases per year since 2003 versus 65 in our study for the same period. These differences could have several nonexclusive reasons. First, diagnosing zygomycosis is not easy. As recently underlined (*25*), a culture positive with a zygomycete does not always mean infection; many gastrointestinal cases reported in the PMSI may be false-positive cases resulting from laboratory contamination. The unusual distribution of clinical presentations found in our study and the fact that one third of clinical information was missing favor this hypothesis. Second, we may have overestimated the number of cases because of undetected duplicates or coding errors. However, inversely, underreporting from the voluntary-based NRCMA network may also occur.

The relevance of hospital discharge data based on ICD-10 codes to estimate the incidence of a rare disease is regularly debated because of limitations in diagnosis accuracy, inconsistent disease coding, or undetected duplicates because of anonymous data recording (*26*)*.* As reported by Chang et al. (*27*), who compared administrative data with medical records in the United States, the positive predictive value of ICD-10 codes for detecting invasive aspergillosis can be low. The authors stressed that these codes may sometimes be assigned before final diagnosis is confirmed, or they may not be assigned by the clinician in charge of the patient. These considerations are also true in the PMSI: overreporting was estimated at 11% for cancers of the thyroid in a study based in 10 districts (*28*) and at 15% for mesothelioma (*29*). Inversely, underreporting caused by diagnostic or coding errors was estimated at 17% by Hafdi-Nejjari et al. (*30*) and at 27% by Carré et al. (*28*), respectively. Nevertheless, administrative data are useful tools to estimate the trends of severe infections like zygomycosis, since most patients are likely to be hospitalized (*26**,**31*)*,* especially in the tertiary care centers that participate in the PMSI.

We were able to document that the incidence of zygomycosis increased in the population of patients with hematologic malignancies or BMT, as already noticed in the 1990s (*32*). In this population, increases in the number of recorded cases could be linked to frequent prescription of antifungal drugs lacking activity against zygomycetes, such as voriconazole and caspofungin (*10**,**17**,**18**,**33**–**35*)*.* However, the role of previous exposure to these antifungal drugs does not appear exclusive because the incidence also increased in the population of patients with diabetes mellitus, a population not known to be commonly exposed to long-term antifungal therapies. An increasing number of the populations at risk (increasing incidence or prolonged survival times) could explain the increase observed in our study: +24% per year for patients with hematologic malignancies and +15% for BMT patients, respectively. However, data from the national agency in charge of the surveillance of transplantations (*36*) indicate that the number of hematopoietic stem cell transplantations increased by +1.58% per year (p<0.01) 2001–2006*.* Similarly, we documented a +9% per year increase in zygomycosis in patients with diabetes; in parallel, diabetes prevalence increased at an estimated 5.7%/year in France from 2000 through 2005 (*37*). We thus tend to consider that the observed trends reflect a genuine increase of zygomycosis cases in these at-risk populations.

### Patients’ Characteristics

The PMSI provides scarce information about the sociodemographic characteristics of patients. In a review of 929 cases published from 1940 through 1999, Roden et al. (*8*) indicated a 1.8 M:F sex ratio and an average age of 38.8 years, versus 1.1 M:F sex ratio and 57.1 years in our study, respectively. However, age distributions and underlying conditions are likely to differ between places and to evolve over time, thus not allowing strict comparisons. Another limitation of the PMSI is the absence of information regarding the circumstances of exposure and portal of entry. The highest incidence rates found in 5 rural districts and not in others of comparable age and gender distributions may indicate a particular exposure to molds in rural environments (for instance, a professional exposure) or an increased awareness of these diseases among clinicians and microbiologists/mycologists of the corresponding regions.

### Patient CFRs

Reporting biases may also partially explain the overall low case-fatality ratio in our study: 26.9% in our highest estimate versus 54% in Roden’s review of 929 cases (*8*). Nevertheless, the CFRs for patients with severe immunosuppressive conditions (Table) are close to values reported in the literature. The CFRs in our study are population-based, while reports from the literature mostly refer to large but relatively old series or to series of immunocompromised patients with severe underlying diseases whose outcome is expected to be worse than in the nonimmunocompromised general population (*8*,*32*,*38*). Also, the CFRs in these other studies may include the long-term outcomes for more discrete time frames, for example, 1 year survival rates (*32*), information not available through our type of study. Deaths occurring during hospitalization are not systematically reported in the PMSI, and those occurring outside the hospital may be underdiagnosed in the CepiDc because many practitioners are not aware of zygomycosis. False-positive cases (for instance, zygomycetes culture without infection) can also influence CFR calculations by increasing the denominator. The wide range of CFR estimates calculated with the capture–recapture method reflects these uncertainties.

Recent progress in early diagnosis and treatment strategies over time should also be considered. For instance, the CFR of rhinocerebral forms may vary from 20% to 69% (*16**,**39*)*.* Roden et al. showed a decrease of the global CFR from 84% to 47% over time since the 1950s (*8*). Antifungal drugs combined with surgical resections play an important role in decreasing lethality for localized infections. The CFR reportedly decreased from 70% in patients treated with antifungal drugs alone down to 14% in patients who receive combined antifungal drugs and surgical treatment (*40*).

## Conclusion

Given the stable increase of incidence over years especially among patients presenting known risk factors (Figure 1), we tend to consider that the increasing numbers of zygomycosis cases are likely to reflect the actual temporal trends of zygomycosis infections diagnosed in the French public and private hospitals. Despite the limitations linked to the sources of data we used, this study provides a useful overview of the zygomycosis incidence trends over a decade at a western European country level. It stresses the growing frequency of this severe infection and the need to increase awareness among clinicians of early diagnosis and treatment, especially for patients with hematologic malignancies, BMT, and/or diabetes. As stated by Chang et al. (*27*), administrative data can be regarded as useful screening tools that should be completed by medical record investigation. In fact, our study has represented an opportunity to launch in 2008 a retrospective study of zygomycosis cases identified through the PMSI and the NRCMA*.* This study will provide a description of zygomycosis cases and their outcome in France. In addition, it should enable us to evaluate the predictive value of administrative data for this rare disease.
